# The development of standard samples with a defined number of antigen-specific T cells to harmonize T cell assays: a proof-of-principle study

**DOI:** 10.1007/s00262-012-1351-0

**Published:** 2012-09-18

**Authors:** Satwinder Kaur Singh, Bart Tummers, Ton N. Schumacher, Raquel Gomez, Kees L. M. C. Franken, Els M. Verdegaal, Karoline Laske, Cécile Gouttefangeas, Christian Ottensmeier, Marij J. P. Welters, Cedrik M. Britten, Sjoerd H. van der Burg

**Affiliations:** 1 Department of Clinical Oncology, Leiden University Medical Center, Building 1, K1-P, P.O. Box 9600, 2300 RC Leiden, The Netherlands; 2 Division of Immunology, The Netherlands Cancer Institute, Amsterdam, The Netherlands; 3 Department of Infectious Diseases, Leiden University Medical Center, Leiden, The Netherlands; 4 Department of Immunology, University of Tübingen, Tübingen, Germany; 5 University of Southampton, Southampton, UK; 6 Department of the Translational Oncology, University Medical Center of the Johannes-Gutenberg-University, Mainz, Germany

**Keywords:** Immunomonitoring, HLA/peptide multimer staining, T cells, Immunotherapy, Assay controls

## Abstract

The validation of assays that quantify antigen-specific T cell responses is critically dependent on cell samples that contain clearly defined measurable numbers of antigen-specific T cells. An important requirement is that such cell samples are handled and analyzed in a comparable fashion to peripheral blood mononuclear cells (PBMC). We performed a proof-of-principle study to show that retrovirally TCR-transduced T cells spiked at defined numbers in autologous PBMC can be used as standard samples for HLA/peptide multimer staining. NY-ESO-1_157–165_-specific, TCR-transduced CD8+ T cell batches were successfully generated from PBMC of several HLA-A*0201 healthy donors, purified by magnetic cell sorting on the basis of HLA tetramer (TM) staining and expanded with specific antigen in vitro. When subsequently spiked into autologous PBMC, the detection of these CD3+CD8+TM+ T cells was highly accurate with a mean accuracy of 91.6 %. The standard cells can be preserved for a substantial period of time in liquid nitrogen. Furthermore, TM staining of fresh and cryopreserved standard samples diluted at decreasing concentrations into autologous cryopreserved unspiked PBMC revealed that the spiked CD3+CD8+TM+ T cells could be accurately detected at all dilutions in a linear fashion with a goodness-of-fit of over 0.99 at a frequency of at least 0.02 % among the CD3+CD8+ T cell population. Notably, the CD3+CD8+TM+ cells of the standard samples were located exactly within the gates used to analyze patient samples and displayed a similar scatter pattern. The performance of the cryopreserved standard samples in the hands of 5 external investigators was good with an inter-laboratory variation of 32.9 % and the doubtless identification of one outlier.

**Electronic supplementary material:**

The online version of this article (doi:10.1007/s00262-012-1351-0) contains supplementary material, which is available to authorized users.

## Background

To evaluate immunotherapies for cancer, it is needed to show the specific ability or capacity of the product to achieve a defined biological effect based on its expected mechanism of action. The ultimate measurement of an immunotherapeutic drug is an objective clinical response, but other measurements of its biological activity using a suitable quantitative biological assay that is linked to the relevant biological properties are highly appropriate during the developmental phase. The biological assay must be standardized at the initial phase and according to regulatory guidelines needs to be validated prior to phase III trials if developed as companion diagnostics. Assay validation can be supported by the identification and/or development of reference materials as these reagents are essential for assay calibration, validation and quality control over a period of time. For T cell-based immunotherapies, therefore, the validation of assays that quantify antigen-specific T cell responses is critically dependent on reference samples that contain measurable numbers of antigen-specific T cells. Unfortunately, the availability of such samples is limited. Moreover, lack of a clearly defined number of T cells specific for a defined antigen within each aliquot hampers the assessment of the accuracy of assays applied. Currently, information on the existing frequency of antigen-specific T cells in cell samples is derived from pre-testing in central laboratories or by consensus (i.e., the average frequency as determined by multiple laboratories in inter-laboratory, proficiency panel projects), and this can only serve as an approximation of the real number of antigen-specific cells present in a given sample [1–4]; especially, in the field of cancer immunotherapy, an estimation of frequency may fail to reveal subtle aspects of assay protocols that allow the accurate detection of tumor-specific T cells at the generally low frequencies reported in patients. One of the broadly applied methods to measure and quantify tumor-specific T cells is the use of HLA tetramers (HLA–TM) [5]. In order to generate reference material, it has been proposed to spike PBMC with previously isolated T cell clones (TCC) with known reactivity [6, 7]. However, this is not a robust option because TCC have a limited life span and the production of quality standard samples requires huge expansions from a single TCC, meaning that new TCC (of constant quality) should be generated constantly. To circumvent this problem, others have tried to use T cell receptor (TCR)-transduced cell lines (e.g., K562). However, the outcome of several proficiency panels such as those run by the Immunoguiding Program of the Cancer Immunotherapy Association (CIP) showed that the gating strategy followed to detect antigen-specific T cells has a major impact on the outcome of T cell assays utilizing the flow cytometer [1, 8]. Therefore, we argued that appropriate in-house reference material should not only be handled and tested, but also analyzed in a comparable fashion to materials from clinical trials. Here, we propose to use TCR-transduced T cells spiked into autologous peripheral blood mononuclear cells (PBMC) to overcome these problems. In this proof-of-principle study, we show that such standard samples can stably be preserved in liquid nitrogen before they are used to generate standard curves in which the detection of antigen-specific CD8+ T cells by HLA–TM staining is highly accurate and follows a linear function with high correlations of coefficiency. Using overlay plots, the HLA–TM-positive events observed in the scatter plots of the standard sample are detected exactly within the area as the PBMC from patients. For individual laboratories, TCR-transduced T cells spiked into autologous PBMC may form a valuable tool to increase the accuracy of the measurements of immune responses and for performing periodic quality controls of assays. In addition, they can be used to compare the measurements of different laboratories within proficiency panels, and as such, they can be used to optimize and standardize immunomonitoring assays. Moreover, they offer the possibility to fully compare the results of one laboratory to another.

## Materials and methods

The authors acknowledge the concept of the minimal information about T cell assays (MIATA) framework which was recently published [9, 10]. Detailed information is structured in the 5 modules proposed by MIATA: the sample, the assay, the data acquisition, the data analysis and the laboratory environment in which the T cell assays were performed.

### Media and reagents

IMDM (Lonza, Verviers, Belgium), supplemented with 100 U/mL penicillin/100 μg/mL streptomycin (Invitrogen, Grand Island, NY, USA), 2 mM l-glutamine (Cambrex, East Rutherford, NJ, USA) and 10 % fetal calf serum (FCS; PAA Laboratories, Pasching, Austria), assigned as complete IMDM, or X-Vivo 15 medium (Lonza) were used as indicated. Retronectin used was from Takara (Otsu, Japan). The peptidic epitope of NY-ESO-1_157–165_ (LLMWITQV) was used in this study and was synthesized with >95 % purity [11], dissolved in DMSO at the concentration of 50 mg/mL, further diluted to a concentration of 1 mg/mL in phosphate-buffered saline (PBS) and stored in aliquots at −20 °C. The cytokines used in this study were GM–CSF (800 IU/mL; Immunotools, Friesoythe, Germany), IL-2 (150 IU/mL; (Aldesleukin, Novartis, Arnhem, the Netherlands)) and IL-15 (5 ng/mL Pepro tech, Rocky Hill, NJ, USA). The APC-conjugated NY-ESO-1_157–165_ peptide tetramer was made in the LUMC in-house facility. The antibodies used were CD45-FITC (clone 2D1), CD3-PE (clone SK-7), CD8-FITC (clone SK-1) and CD8-PerCP-Cy5.5 (clone SK1), all purchased from BD biosciences.

### The sample

PBMC used in this study were isolated from HLA-A*0201 anonymous healthy blood bank donors (Sanquin, The Netherlands) after informed consent. PBMC were isolated within 24 h after blood draw by Ficoll density gradient centrifugation, resuspended in FCS, put on ice for 15 min and after dropwise addition of an equal volume of 80 % FCS and 20 % DMSO (Sigma, St. Louis, MO, USA) cryopreserved using a freezing container (Mr. Frosty, Thermo Fisher Scientific, Langenselbold, Germany). Equal aliquots of cells (10^7^/vial) were stored in the vapor phase of the liquid nitrogen vessel until further use. In addition, PBMC from 7 stage IV melanoma patients were isolated from heparinized venous blood and stored in liquid nitrogen until use. All patients gave written informed consent. The treatment protocol was approved by the Medical Ethics Committee of the Leiden University Medical Center. All patients were HLA-A*0201 positive as determined by HLA genotyping. The expression of NY-ESO-1 on the corresponding tumor cells of the melanoma patients was determined by specific RT-PCR [12]. The handling and storage of the blood samples were performed according to the standard operating procedure (SOP) of the Department of Clinical Oncology, Section Experimental Cancer Immunology and Therapy at the Leiden University Medical Center by well-trained personnel.

### Virus production

The packaging cell line FLY-RD18 was used to produce retroviruses encoding NY-ESO-1-specific TCR. Briefly, the HLA-A*0201 restricted NY-ESO-1_157–165_-specific TCR construct (the full-length codon-optimized TCR AV23.1 and TCR BV13.1 [13]) chain sequences of a dominant TCC of patient LAU 155 [14] was used. At day 0, FLY-RD18 were seeded at the density of 1.5 × 10^5^ cells per well in 6-well plate in 2 mL of complete IMDM. At day 1, cells were transfected with 2.5 μg retroviral vector DNA-encoding NY-ESO-1 TCR using transfection reagent Fugene6 (Roche) according to manufacturer’s protocol. After 48 h, the virus-containing supernatant was collected, centrifuged at 2,000 rpm for 8 min, aliquoted and snap-frozen. Viruses were stored at −80 °C until further use.

The transfected FLY-RD18 were trypsinized, washed with cold PBS/0.5 % bovine serum albumin (BSA) and fixed with 4 % paraformaldehyde (PFA) on ice for 4 min. Cells were intensively washed and then permeabilized with PBS containing 0.5 % BSA, saponin and 10 % FCS for 10 min on ice. Cells were then washed and stained with PE-conjugated anti-TCR Vβ13.1 (NY-ESO-1-specific TCR Vβ chain) in dark on ice for 30 min. After 30 min, cells were washed twice, fixed with 1 % PFA and acquired by FACS Calibur (BD biosciences, USA, CellQuest Pro). The analysis of the flow cytometry standard (FCS)-format data files was performed by using FlowJo (Tree Star Inc., OR, USA).

### Transduction of PBMC

At d0, PBMC were thawed, counted and resuspended in X-Vivo 15 medium containing 0.5 μg/mL mitogen Phytohaemagglutinin HA-16 (PHA; Remel Europe) and IL-2 (20 U/mL) at a concentration of 1 × 10^6^ cells/mL and cultured in 24-well plate for 36 h at 37 °C, 5 % CO_2_ and 92 % humidity. At day 1, a 24-well culture plate (Falcon) was coated with 0.5 mL/well of 50 μg/mL retronectin in PBS (Takara, Otsu, Japan) overnight at 4 °C. On day 2, retronectin was removed and wells were blocked for 30 min at room temperature (RT) with PBS/2 % BSA. After 30-min incubation, wells were washed with PBS and incubated with 0.5 mL supernatant containing virus particles/well at 37 °C for 2–3 h. Then, the PHA-activated PBMC were harvested and washed with PBS + 0.5 % BSA + 2 mM EDTA (EDTA buffer) and CD4+ T cells were depleted using Dynal beads according to manufacturer’s protocol (Invitrogen). The enriched CD8+ T cell fraction was counted, washed and resuspended in warm X-Vivo 15 medium at a concentration of 1 × 10^6^ cells/mL. The enriched CD8+ T cells (0.5 × 10^6^ cells/well) were added to the retronectin-coated plate containing virus, and the plate was centrifuged at 2,000 rpm (acceleration 3, deceleration 0) for 90 min. Cells were then cultured at 37 °C for 2 days. On day 5, transduced cells were harvested, counted and washed with EDTA buffer. Dead cells were removed by spinning cells on 4–5 mL Ficoll at 2,000 rpm for 15 min. Cells obtained from the interphase were washed twice with EDTA buffer and incubated with dynal beads to remove CD4+ T cells according to manufacturer’s protocol. After purification, the cells were washed and stained with APC-conjugated NY-ESO-1_157–165_ peptide tetramer at RT, 30 min in the dark, washed, then resuspended in EDTA buffer plus 20 μL anti-APC microbeads (Miltenyi Biotec, Germany) and incubated in the dark at 5–8 °C for 15 min. Subsequently, the cells were washed with EDTA buffer and resuspended in 0.5 mL cold EDTA buffer, after which the cells were purified on two successive MS column as per manufacturer’s protocol. Purified cells were stained with anti-CD8-FITC and CD3-PE antibodies to assess purity of NY-ESO-1-specific TCR-expressing CD3+CD8+ T cells, cells were analyzed using FACS Calibur (CellQuest Pro), and the FCS files were analyzed by FlowJo.

Purified cells were clonally expanded by co-culturing them with NY-ESO-1 CTL epitope–loaded GM–CSF-activated autologous plastic adherent monocytes preloaded with 5 μg/mL NY-ESO-1_157–165_ peptide for 3–4 days in X-Vivo 15 containing 150 U/mL IL-2 and 5 ng/mL IL-15 in flat-bottom plates. After 3–4 days, T cells were harvested, medium was refreshed, and the cell cultures were split if required and cultured in round-bottom plates until the cells started to round off; this was considered the appropriate moment to generate the standard sample by spiking desired percent of NY-ESO-1-specific TCR-expressing CD3+CD8+ T cells into autologous PBMC.

### The assay, acquisition and analysis of PBMC and spiked standard samples for antigen-specific T cells

To analyze the number of cells expressing the NY-ESO_157–165_ peptide–specific TCR, cells were stained first with the HLA–TM (25 μL/sample; 1:100 diluted in EDTA buffer) for 30 min at RT, washed with 100 μL EDTA buffer, divided into two wells and then stained (25 μL/sample) either with antibodies (diluted in EDTA buffer) to CD3 (PE labeled; 1:10) and CD8 (PerCP-Cy5.5 labeled; 1:30) or with antibodies to CD45 (FITC labeled; 1:200) for 20 min at 4 °C. The cells were then washed twice and fixed with 1 % PFA. The stained samples (at least 25,000 cells per sample) were acquired by FACS Calibur flow cytometer (software CellQuest Pro). Analysis of the FCS files was performed using FlowJo.

### Proficiency panel

In order to test the performance of our standard sample in the hands of external investigators, a proficiency panel was run by the CIP, designated as CIP_ID10_2010_MUL/E. The participating laboratories received a dry ice package containing 2 vials of TCR-transduced cells—spiked at 0.25 %—in autologous PBMC (5 × 10^6^) and an aliquot of 5 μL APC-labeled NY-ESO_157–165_ peptide tetramer. The laboratories were asked to perform their analyses within 7–10 days after the package had arrived. The laboratories were instructed to first stain with the HLA/peptide tetramer (1:100) 30 min at RT, to wash the cells and then to perform CD3–CD8 or CD3–CD45 staining for 20 min at 4 °C. They were required to fix the cells directly after staining for safety reasons as part of the cells express retrovirally transduced TCR. Along with these instructions, they received the flow cytometer plots with the gating strategy as generated by the Laboratory of Clinical Oncology of the LUMC, when analyzing the standard sample freshly after spiking, as a guide for their own analysis. The laboratories were asked for their staining protocol, the antibodies used (clone, company, dilution) and the type of flow cytometer used besides their results (FACS plots).

### Statistical methods

Mean, standard deviation (SD) and coefficient of variation (CV) are shown for experiments when required. The accuracy of detection was calculated by the following formula: (the percentage of detected HLA–TM+ cells/the percentage of expected HLA–TM+ cells) × 100.

### Laboratory environment

The Laboratory of the Clinical Oncology, Section Experimental Cancer Immunology and Therapy at the Leiden University Medical Center, is a research laboratory where the assays are performed according to SOPs, including the predefined criteria for positive responses, by well-trained personnel. The laboratory—as well as those participating in the small proficiency panel described here—has participated in all proficiency panels on HLA tetramer analysis of the CIMT immunoguiding program (CIP; http://cimt.eu/workgroups/cip/proficiency-panel-program/).

## Results

### Successful production of tumor-antigen-specific TCR-transgenic CD8+ T cells using healthy donor peripheral blood mononuclear cells

**Fig. 1:**
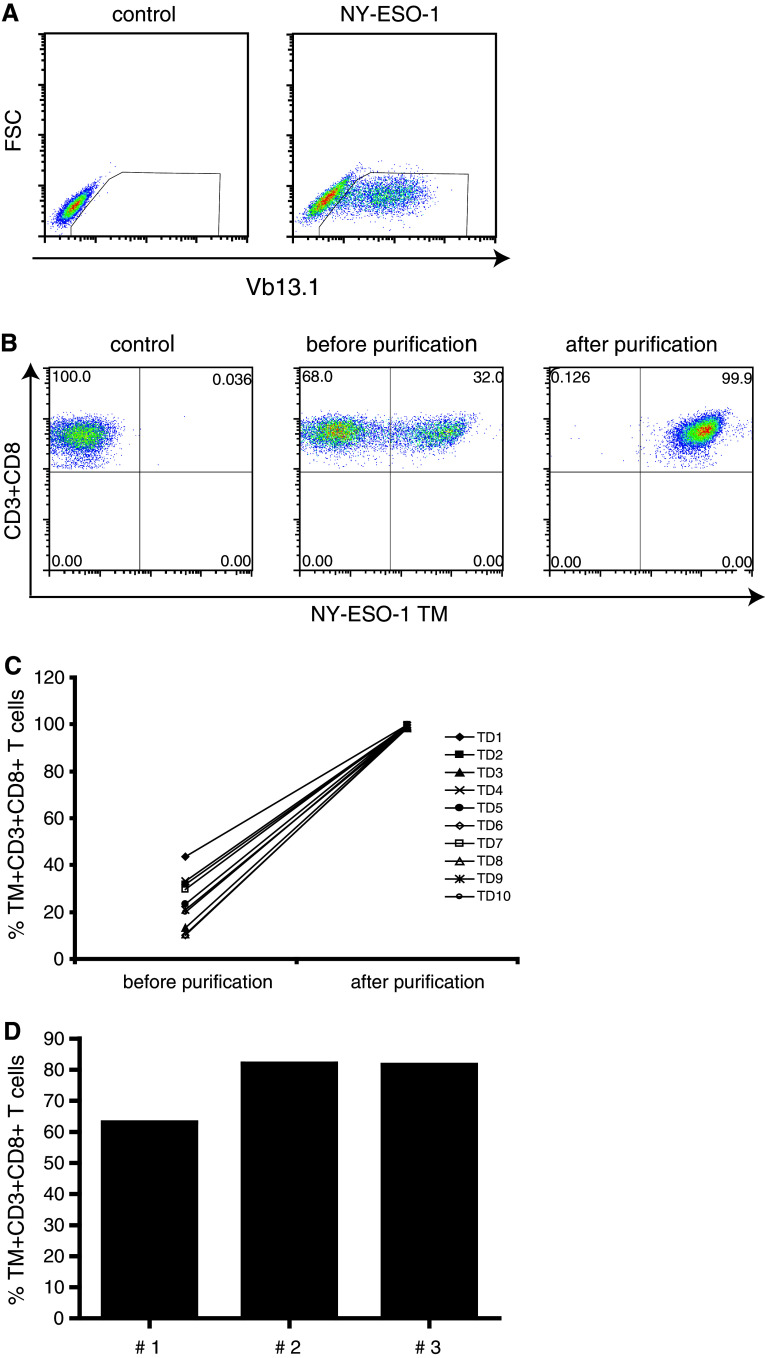
Generation of standard samples. A standard sample was generated by introduction of NY-ESO-1-specific TCR into activated CD8+ T cells. **a** A retrovirus expressing the NY-ESO-1-specific TCR was produced by transfecting the FLY-RD18 packaging cell line with the NY-ESO-1 constructs. The intracellular detection of TCRVβ13.1 indicates the introduction and expression of the TCR construct in FLY-RD18 cell line. As control, non-transfected FLY-RD18 cells were used. **b** The activated CD8+ T cells were transduced with this recombinant retrovirus, and after 2 days, the transduced cells were harvested, labeled with NY-ESO-1-specific tetramers and then purified by magnetic cell sorting. **c** The purification process resulted consistently in populations with a purity higher than 98 % [*n* = 10 test donors (TD)]. **d** The purified CD8+ T cells expressing NY-ESO-1 TCR were clonally expanded by using peptide-pulsed autologous monocytes and analyzed for purity after 2 weeks of culture. Data from three different transductions with PBMC of different donors are shown

We aimed at generating standard samples with defined number of antigen-specific CD8−T cells spiked into autologous PBMC for use in HLA/peptide multimer experiments following a similar gating strategy as applied to patient PBMC samples. As model, we chose the tumor-antigen NY-ESO-1 because the frequency of NY-ESO-1-specific T cells is generally very low in PBMC from healthy donors [6]; hence, it would be possible to assess the detection of low frequencies of NY-ESO-1-specific T cells when spiked into autologous PBMC. We used a HLA-A*0201-restricted NY-ESO-1_157–165_-specific TCR construct (the full-length codon-optimized TCR AV23.1 and TCR BV13.1 [13] chain sequences of a dominant TCC of patient LAU 155 [14] which was transfected in FLY-RD18 packaging cell line for the production of retroviruses encoding this TCR. As a control for transfection of the packaging cells, the expression of the TCRVβ13.1 chain was analyzed (Fig. 1a). PBMC from 10 HLA-A*0201 healthy donors were transduced using the engineered retrovirus. In each experiment, on average, 30 million PBMC were activated using PHA and IL-2. The CD8+ T cells were isolated and subsequently retrovirally transduced with the HLA-A*0201-restricted NY-ESO-1-specific TCR. On average, 24 % (range 18–42 %, *n* = 10) of the CD8+ T cells expressed the transgenic TCR. In order to obtain a pure population of HLA-A*0201-restricted NY-ESO-1-specific CD8+ T cells with proven capacity to bind HLA–TM, the TCR-transgenic T cells were stained with HLA-A*0201 NY-ESO-1_157–165_ TM and then isolated by magnetic sorting (Fig. 1b). Purification of different batches of transduced cells consistently resulted in highly pure (>98 %) populations of NY-ESO-1-specific TCR-expressing CD8+ T cells (Fig. 1c); however, the yield was low (mean = 7 × 10^5^, SD = 2 × 10^5^ and CV = 25 %) but consistent across different transduced T cell batches. To obtain a higher number of antigen-specific TCR-transgenic CD8+ T cells, the freshly transduced T cell fractions were subjected to one cycle of antigen-specific expansion using autologous monocytes pulsed with the NY-ESO-1_157–165_ peptide. After 2 weeks, the expanded T cell populations contained 65–80 % TCR-transgenic NY-ESO-1-specific CD8+ T cells as measured by HLA–TM analysis (Fig. 1d). These results show that the NY-ESO-1-specific TCR can be successfully introduced into PBMC of healthy donors, that the transgenic TCR is constitutively expressed at a high level by the TCR-transduced CD8+ T cells, and that these TCR-positive T cell fractions can be isolated and expanded reproducibly for further use.

### Accuracy and stability of standard samples with a defined percentage of transgenic TCR-expressing CD8+ T cells

**Fig. 2:**
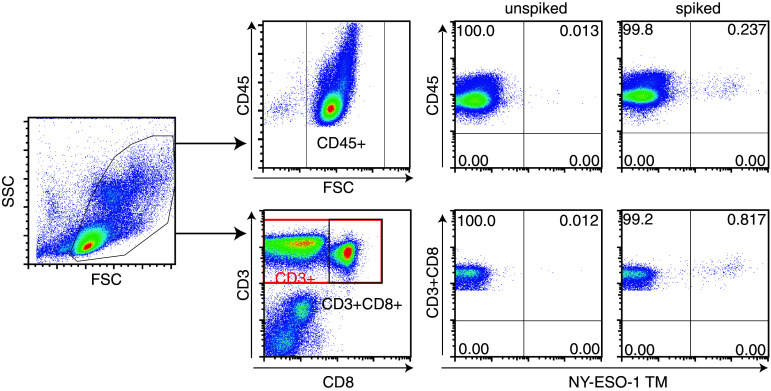
Gating strategy for analyzing standard samples. The standard sample was prepared by spiking a defined percentage (0.25 %) of antigen-specific TCR-transduced T cell batch into autologous PBMC. Cells were stained for TM, CD3 and CD45 or CD8 and analyzed by flow cytometry. Live PBMC were gated on *forward* (FSC) and *side* (SSC) *scatter*. A subsequent gate was set at CD45-FSC or the CD3+CD8+ population. Then, either the CD45+ or the CD3+CD8+ population was plotted against NY-ESO-1-specific TM. Both the unspiked and the spiked PBMC samples are shown

After the successful generation of transgenic NY-ESO-1-specific TCR-expressing CD8+ T cells, we aimed to create standard samples with a defined percentage of antigen-specific CD8+ T cells and to test whether it is possible to retrieve that percentage by TM staining and flow cytometry. A batch of TCR-transgenic CD8+ T cells was generated that after expansion contained 94.2 % of TM-positive CD8+ T cells. This population of T cells was spiked as a 0.25 % population in autologous PBMC, stained with TM and surface antibodies to CD3 and CD8 or CD45 and analyzed by flow cytometry (Fig. 2). Viable PBMC was gated based on forward and side scatter. The TCR-transduced T cells were spiked as a percentage of the total PBMC and not only T cells; therefore, we analyzed the percentage of CD45+ NY-ESO-1 TM+ cells as the detected percentage should be close to the spiked percentage of cells. Furthermore, the CD3+CD8+TM+ cells were analyzed (Fig. 2). The exact percentage of expected NY-ESO-1-specific CD8+ T cells spiked into CD45+ autologous PBMC was calculated by multiplying the measured percentage of HLA–TM-positive TCR-transduced CD8+ T cells (94.2 %) with the percentage of spiked cells (0.25 %), resulting in an expected percentage of 0.24 % of NY-ESO-1-specific T cells within the total (CD45+) PBMC population. The percentage of detected TM+ cells in the CD45 population in the spiked PBMC sample was 0.237 % CD45+TM+ cells and in the non-spiked PBMC sample 0.013 %. Subtraction of this background indicates that the exact percentage of detected spiked cells was 0.224 %, which was close to what was expected. Similarly, the percentage of expected TM+ spiked CD8+ T cells among the CD3+CD8+ cell population in PBMC was calculated to be 0.93 % of TM+CD3+CD8+ T cells. For this, the percentage of viable cells was divided by the percentage of cells in the CD3+CD8+ gate and multiplied by the expected percentage of spiked cells among the CD45+ population (94.8 % live cells/24.3 % CD3+CD8+ cells × 0.24 % CD45+ HLA–TM+ cells = 0.93 %). We detected 0.817 % TM+ cells within the CD3+CD8+ autologous PBMC populations, which was corrected for background, resulting in the detection of 0.805 % CD3+CD8+TM+ spiked cells. Similar results were obtained with 6 other standard cell samples that were generated (Online Resource 1), revealing that the detection of the spiked cells was highly accurate when gated on CD45+ cells (mean accuracy 99.6 %; range 97–117 %) and when gated on CD3+CD8+ T cells (mean accuracy 91.6 %; range 78–113 %).

**Fig. 3:**
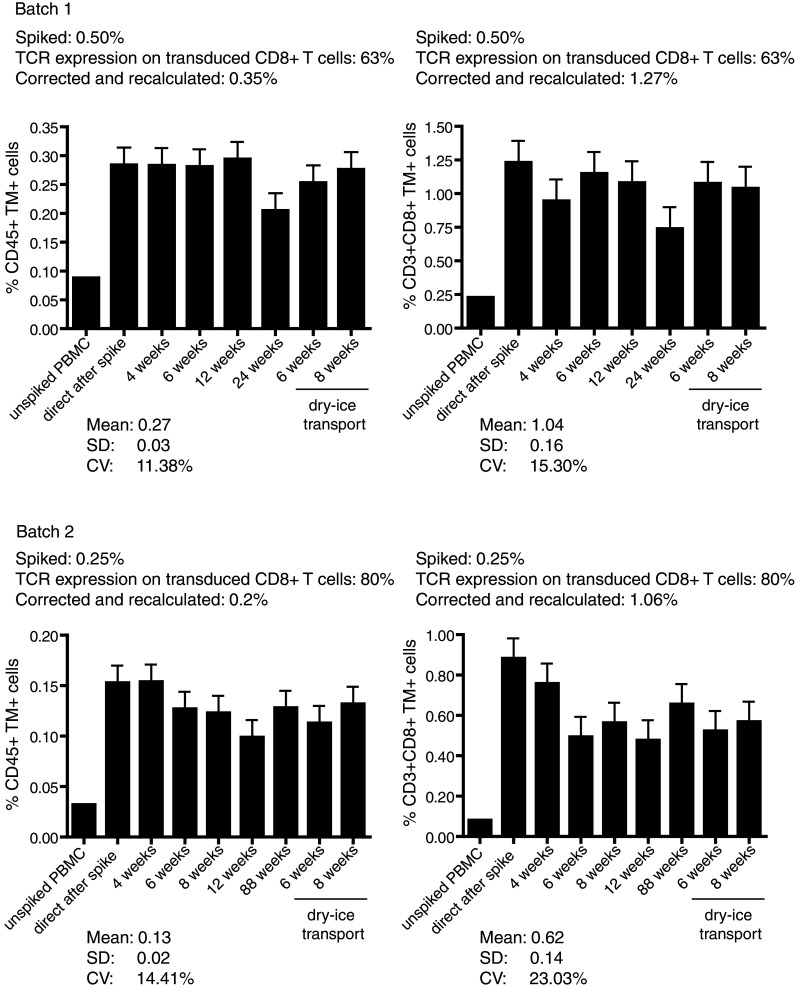
Stability of standard samples over time. Two batches of standard samples were prepared and analyzed for the percentage of NY-ESO-1-specific CD45+ T cells and CD3+CD8+ T cells over a period of time (batch 1: 0–24 weeks; batch 2: 0–88 weeks). In addition, samples, after 5 weeks after storage in liquid nitrogen, were taken home for overnight and brought back on dry ice transport and then replaced in liquid nitrogen again. Around 1 and 3 weeks later, they were analyzed with respect to the frequency of the NY-ESO-1-specific T cells. The mean, standard deviation (SD) and coefficient of variance (CV) are depicted for both batches

After having established that, it is possible to detect a defined percentage of antigen-specific T cells when spiked into autologous PBMC within acceptable limits we set out to test the stability of standard samples stored in liquid nitrogen over time. For this, we used two batches of TCR-transduced CD8+ T cells. After expansion, the percentage of CD8+TM+ T cells for batch 1 was 63 %, and for batch 2, it was 80 % (data not shown). Batch 1 was spiked at a concentration of 0.50 %, and batch 2 was spiked at a concentration of 0.25 % in their respective autologous PBMC. The expected percentages of the TM+CD3+CD8+ T cell populations were calculated, indicating that for batch 1 0.32 % (i.e., 0.63 × 0.5) of the CD45+ PBMC and 1.27 % of the CD3+CD8+ T cells in the PBMC should stain with the TM (84.45/20.9 × 0.32). In the batch 2 spiked PBMC, it was expected to detect 0.20 % (0.8 × 0.25) of TM+ cells among CD45+ PBMC and 1.06 % (94.90/17.96 × 0.2) TM+ cells within the CD3+CD8+ gate. After subtraction of background measured in the non-spiked PBMC, we specifically detected 0.284 % TM+ cells and 0.153 % TM+ cells among the CD45+ PBMC population, respectively, in batch 1 and batch 2 of freshly spiked cells. In order to determine the stability of the standard samples when frozen and stored in liquid nitrogen, the samples were stored and aliquots of the cryopreserved samples were subsequently thawed and analyzed at different time points (Fig. 3). Long-term storage (>24 weeks) did not result in a significant changes as the percentages of TM+ cells detected over time were well within the range—as indicated by the low covariance being <16 % for batch 1 and <24 % for batch 2—of what was detected at earlier time points. To mimic transportation conditions, the standard samples were transferred from the liquid nitrogen to dry ice for 1–2 days and then back to liquid nitrogen again. The temperature of dry ice was not monitored during shipment. Transportation of standard samples on dry ice did not negatively affect the percentage of TM+ cells detected afterward (Fig. 3).

### Cryopreserved standard samples are useful for the accurate detection of antigen-specific T cells present at low frequencies

**Fig. 4:**
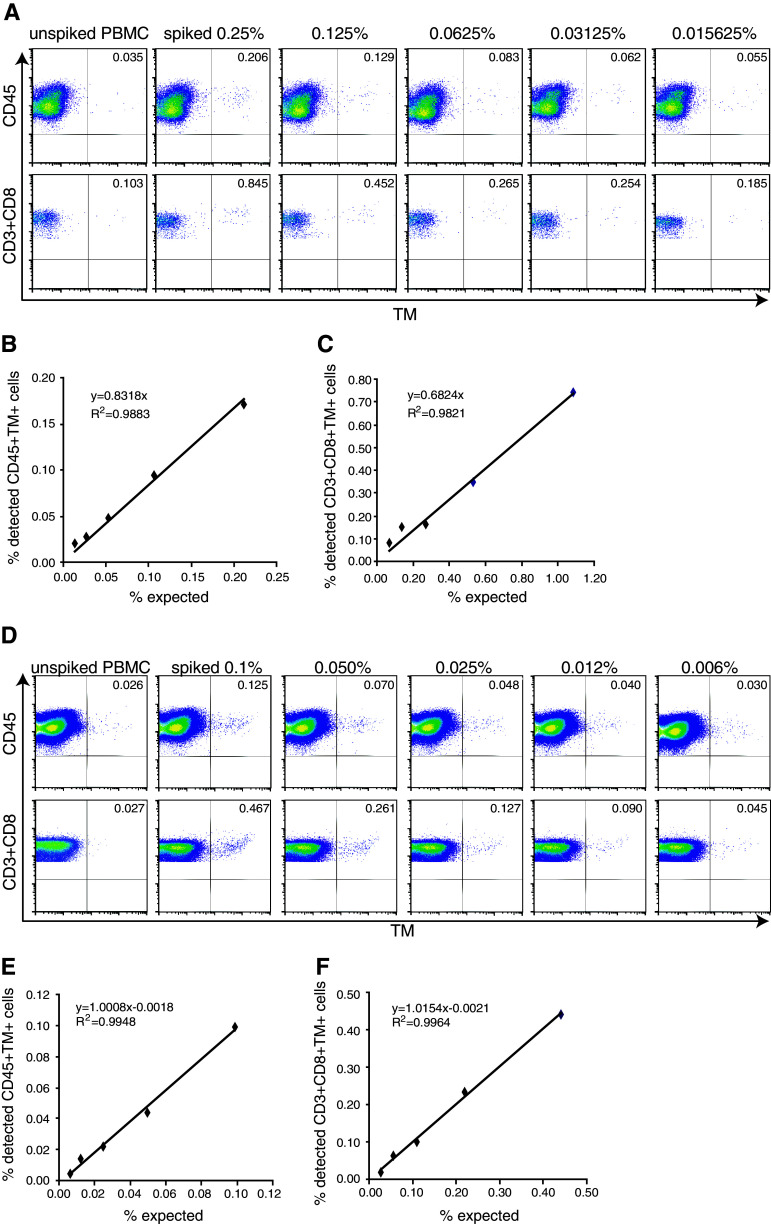
Accuracy and linearity of detection is high when fresh or frozen standard cells are used to make a standard curve. Autologous PBMC were spiked at the indicated percentages with NY-ESO-1-specific TCR+ cells and stained for CD45 or CD3 and CD8 and the NY-ESO-1-specific TCR (by HLA tetramers). **a** A representative analysis of a standard curve generated with freshly isolated NY-ESO-1-specific TCR-transduced CD8+ T cells. The flow cytometry plots show either the CD45+ PBMC population or the CD3+CD8+ PBMC population. To determine the accuracy and linearity of detection, the percentage of measured CD45+TM+ cells (**b**) or CD3+CD8+TM+ cells (**c**) minus the background measured in the unspiked PBMC sample was *plotted* against the expected percentages of CD45+TM+ or CD3+CD8+TM+ cells. Linear regression analysis was used to determine a *line* through all data points, revealing a high goodness-of-fit as demonstrated by a coefficient of determination close to 1. **d**–**f** A similar analysis was performed as in **a**–**c,** but here the standard curve is made by using a standard sample (0.25 % spiked in autologous PBMC) that had been stored in liquid nitrogen. The sample was thawed and diluted into thawed autologous PBMC just before analysis at the indicated percentages

Optimally, the standard sample should allow the accurate detection of antigen-specific T cells present at frequencies similar to that expected in PBMC samples of patients with cancer. To investigate this, we used a batch of TCR-transduced CD8+ T cell with a purity of 84.7 % CD8+TM+ T cells. The batch was spiked in a range of 0.25–0.015 % cells into autologous PBMC. The freshly spiked samples were stained with TM and anti-CD3 with either anti-CD45 or anti-CD8 and analyzed by flow cytometry. The highest expected percentage of CD45+TM+ cells in the PBMC sample was calculated at 0.21 % (0.847 × 0.25), and 0.171 % spiked CD3+CD8+TM+ T cells were detected based on the determined frequency of 0.206 % TM+ cells and the background TM staining in the naive PBMC sample (Fig. 4a). The percentages of detected CD45+TM+ cells or CD3+CD8+TM+ T cells were plotted—after subtraction of the background detection in the native (non-spiked) sample—against the expected percentages of TM+ cells among the respective populations. The detection of spiked TM+ cells followed a linear function with a high correlation coefficient across all the high to very low tested frequencies as shown by the linear regression analysis displaying a high goodness-of-fit (Fig. 4b, c; *R* ^2^ > 0.98), suggesting that even small increases in the percentage of TM+ can be correctly detected.

The generation of standard samples is most useful when one is able to store these cells for later use. Therefore, PBMC spiked with very low to high frequencies of TCR-transduced CD8+TM+ T cells were cryopreserved and thawed after 4 weeks of storage. Analysis of these samples revealed a loss of CD3+CD8+TM+ T cells that was specifically noticeable in the samples spiked with low frequencies of NY-ESO-1-specific T cells and reflected by a failure to detect low-frequency T cells and a loss of the linearity of detection at the lower frequency range. The lowest accurate measurement of TM+ cells in the CD45 population increased to 0.055 % and that of TM+ cells in the CD3+CD8+ population to 0.20 % (data not shown). Therefore, an alternative approach was chosen in which a cryopreserved standard sample containing a relatively high concentration (0.25 %) of TCR-transduced CD8+ T cells was thawed and diluted at decreasing concentrations into autologous cryopreserved unspiked PBMC. Analysis of the percentage of TM+ cells in these samples showed that an increase in the percentage of TM+ cells as compared to the unspiked sample could be detected at all dilutions (Fig. 4d). Plots of the detected (minus background of unspiked sample) and expected percentages of TM+ cells in either CD45+ cells (Fig. 4e) or CD3+CD8+ T cells (Fig. 4f) revealed strong linearity with a goodness-of-fit of over 0.99 and the possibility to accurately detect antigen-specific (TCR-transduced) T cells at a frequency of >0.004 % among the CD45+ and at a frequency of >0.02 % in the CD3+CD8+ cell population. This shows that cryopreserved standard samples when diluted in unspiked autologous PBMC directly before analysis can be used to prepare a standard curve enabling a controlled detection of low-frequency antigen-specific cells in patients.

**Fig. 5:**
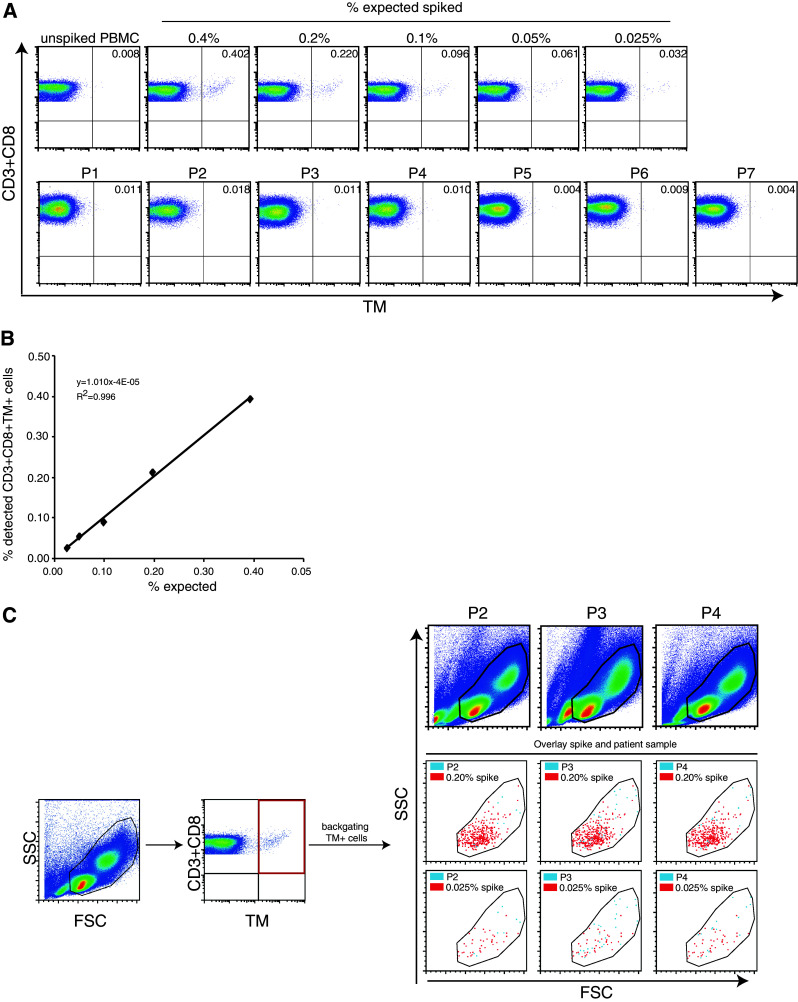
Analysis of standard samples is not different from patient samples. To analyze the usefulness of these standard samples in a day-to-day use, a vial containing a percentage of 0.25 % NY-ESO-1-specific TCR-transduced T cells was diluted at indicated percentages in autologous thawed PBMC and used to generate a standard curve. In addition, the PBMC of 7 different patients with stage IV melanoma were thawed (notably tumors of P2, 3 and 5–7 were NY-ESO-1 positive by mRNA analysis). **a** The standard samples and patient samples were stained for CD3, CD8 and NY-ESO-1-specific TCR by tetramers. None of the patient samples showed a staining by tetramers above the staining observed in the lowest reference sample used. **b** The measured percentages of NY-ESO-1-TM+CD3+CD8+ T cells in the standard samples minus the percentage of *positive* cells in the unspiked sample were *plotted* against the expected percentage of TM+ cells. Linear regression analysis demonstrated good linearity of detection. **c** Back-gating of the tetramer-positive T cells in the standard samples showed their location within the gates used to analyze the standard samples and revealed that they are well placed within the gates one would apply to PBMC of patient samples

We then put this to the test and prepared a standard curve from a cryopreserved standard cell sample as well as tested the PBMC of a number of melanoma patients for the presence of NY-ESO-1-specific T cells by TM staining and flow cytometry. The tumors of patients 2, 3 and 5–7 were found to be NY-ESO-1 positive by mRNA (not shown). The standard curve ranged from an expected 0.025–0.40 % of TM+ cells within the CD3+CD8+ T cell population. The expected percentages of NY-ESO-1-specific TCR-transgenic CD8+ T cells across the whole range of low to high frequencies were accurately detected (Fig. 5a). Again, a clear linear relationship existed between the detected and expected populations of CD3+CD8+TM+ T cells (Fig. 5b). Of note, a twofold increase in immune activity over background often is used to define a positive response [15, 16]; when we plotted the percentages of observed TM+ cells among the CD3+CD8+ T cell population without subtraction of background versus the expected population of CD8+ T cells (Online Resource 2), the data started to become linear at twice the background. Unfortunately, none of the patients displayed a response that exceeded the number of TM-positive events in our lowest standard cell control. We then back-gated the TM-positive events in the control sample, and by overlaying the plots, we showed that the CD3+CD8+TM+ T cells were located exactly within the gates used to analyze patient PBMC samples. In addition, the few HLA–TM+ events in the patient samples and the abundantly present HLA–TM-positive events in the control sample showed the same scatter pattern (Fig. 5c).

### A 5-center proficiency panel validates the standard sample

**Fig. 6:**
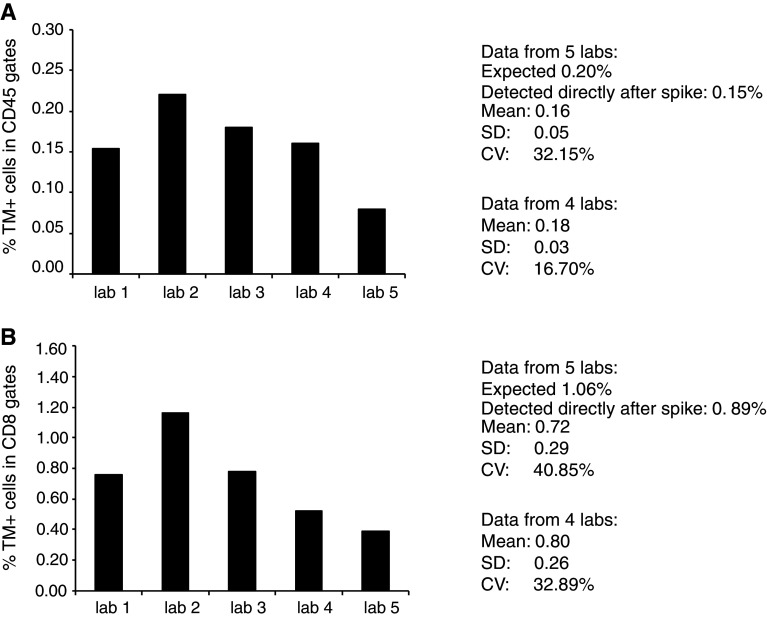
Use of a reference sample in a small 5-center proficiency panel. **a**, **b** A standard sample with a defined number of NY-ESO-1-specific cells (expected 0.2 % CD45+TM+ and 1.06 % CD3+CD8+TM+ cells, measured directly after spike 0.15 and 0.89 %, respectively) was distributed together with the APC-labeled NY-ESO-1_157–165_ tetramer between 5 laboratories. Each laboratory had participated in HLA multimer proficiency panels before and used their own laboratory-specific protocol to analyze the sample. A gating instruction was provided. Data were reported back. Laboratory number 5 was a typical outlier. The mean, SD and CV were calculated for all five laboratories as well as after removal of the outlier laboratory

In order to test the performance of our standard sample in the hands of external investigators, we designed a proficiency panel with 5 selected participants. A standard sample with a defined number of NY-ESO-1-specific cells (expected 0.2 % CD45+TM+ and 1.06 % CD3+CD8+TM+ cells, measured directly after spike 0.15 and 0.89 %, respectively, not shown) was distributed to 5 laboratories. The laboratories were also provided with NY-ESO-1-specific tetramer but were allowed to use their own protocol to analyze the standard sample. Samples were stained for HLA–TM, CD3 and either CD45 or CD8. A guideline for gating was provided in the form of our analysis, showing the flow cytometer plots and gates, of this standard sample when analyzed directly after spiking. Figure 6 depicts the results from this proficiency panel. We observed that NY-ESO-1-specific TM+ cells were detected by all five laboratories, albeit that laboratory 5 detected a much lower percentage of TM+ cells when compared to the other four laboratories. These four laboratories detected the NY-ESO-1-specific TM+ cells among the CD45+ cells at a similar frequency as reflected by a low covariance value of 16.70 % (Fig. 6a) and with a mean frequency (0.18 %) close to the expected (0.20 %) and to the frequency of TM+ cells measured directly after spiking (0.15 %). The detection of NY-ESO-1-specific TM+ cells within the CD3+CD8+ cell gate was also similar in those 4 laboratories with a mean frequency of 0.80 % versus the fresh measured frequency of 0.89 % CD3+CD8+TM+ cells resulting in an inter-laboratory variation of 32.8 % (Fig. 6b). These data show that it is not only possible to successfully run a proficiency panel with cryopreserved standard samples but also now possible to single out true outliers.

## Discussion

We have performed a proof-of-principle study to show that retrovirally transduced TCR-transgenic T cells can be used as standard samples for the controlled measurement of antigen-specific T cells in patient samples by HLA multimer staining and flow cytometry analysis. Our results show that the method to generate standard samples by retroviral transduction of PBMC followed by HLA tetramer–specific isolation and antigen-specific expansion reproducibly results in high-quality standard samples that can be made with a standard PBMC batch (Fig. 1). When spiked into autologous PBMC, the activated TCR-transduced T cells appear at the exact region (i.e., gates) where one would expect to find resting and activated antigen-specific T cells in patient PBMC samples (Fig. 5), allowing researchers to use the same gating strategy for the standard and test samples during analysis. Whereas it was previously not possible to establish the accuracy of HLA multimer analysis [17], the measurement of spiked samples and standard curves with TCR-transduced T cells in autologous PBMC showed that the detected frequencies of antigen-specific T cells were accurate (Online Resource 1) and well within the range of what is considered to be an acceptable accuracy [17]. Moreover, the detection of spiked TM+ cells followed a linear function with a high correlation coefficient across all the high to very low tested frequencies, indicating that even small increases in the percentage of TM+ can be correctly detected (Figs. 4, 5). The intra-assay precision was also acceptable with CV < 25 % as determined by the measurement of samples that were stored for the short and long term in liquid nitrogen before analysis (Fig. 3). In addition, our standard curve showed that the lower limit of accurate detection is 0.02 % of CD8+ T cells, which is within the range of limits of detection (0.01–0.04 %) established by others using HLA tetramers for HLA-A2-restricted CD8+ T cells epitopes in gp100, MART, HIV, CMV and influenza [17–20].

We performed a small proficiency panel with our standard samples. The laboratories involved had previously participated in the CIP proficiency panels and were harmonized with respect to HLA multimer analysis [1, 21]. The current proficiency panel revealed the capacity of harmonized laboratories to detect a fixed number of antigen-specific T cells with low variation. It also showed that this type of standard samples can be used to identify true outliers, as the frequency of the antigen-specific cells is known. Thus, the standard sample resolves a recurrent discussion point accompanying the evaluation of the outcomes of proficiency panels to date.

Taken together, these features qualify the retrovirally TCR-transduced T cells spiked into autologous PBMC as excellent standard samples that may be used: (a) to validate one’s HLA/peptide multimer assay, (b) as control sample for the analysis of samples at different time points or laboratories within one or multiple trial(s), (c) to identify protocol parameters and the outcome of measurements and (d) to identify protocols/laboratories with a true high or low performance. Provided that the cloned TCRs are available for transduction, the cost to generate standard samples is low, certainly when compared to the costs associated with immunomonitoring or the general costs of a clinical trial. Notably, as these standard samples consist of retrovirally transduced TCR-expressing T cells spiked back into autologous healthy donor PBMC, they form a widely applicable tool. The downside is that use of retroviral TCR transduction requires specialized laboratories to produce standard samples. Although after purification and expansion the cells are free of infectious virus, not all institutes may allow the manipulation of such cells in their flow cytometry facilities or in standard laboratories despite fixation with paraformaldehyde. Furthermore, the organization of proficiency panels may be prohibitively costly when these samples are sent to different countries. A solution to this would be the use of modified RNA as format for delivery of TCR chains to primary lymphocytes, which we are currently exploring, as it may offer a simple, virus-free and scalable process for the manufacturing of reference samples in peripheral laboratories. Overall, our study opens the possibility to engineer standard samples that can be used in a similar fashion as for patient samples to accurately measure the frequency of antigen-specific T cells by HLA/peptide multimer analysis.

### Electronic supplementary material

Below is the link to the electronic supplementary material.

Supplementary material 1 (PDF 278 kb)
