# Supplementary material for: The development of standard samples with a defined number of antigen-specific T cells to harmonize T cell assays: a proof-of-principle study
Source: Cancer Immunol Immunother. 2012 Sep 18;62(3):489–501. doi: 10.1007/s00262-012-1351-0 (PMC3589624; doi:10.1007/s00262-012-1351-0)
Supplement: Supplementary file 1 — Supplementary material 1 (PDF 278 kb) [file 262_2012_1351_MOESM1_ESM.pdf]

CD45+ TM+ cells detected

bg	expected	detected	detected-bg	accuracy
0,09	0,32	0,37	0,28	117,46
0,03	0,20	0,18	0,15	90,00
0,01	0,24	0,24	0,22	100,64
0,03	0,37	0,35	0,33	95,03
0,03	0,19	0,18	0,16	97,18
0,04	0,21	0,21	0,17	97,28

CD3+CD8+TM+ cells detected

bg	expected	detected	detected-bg	accuracy
0,23	1,31	1,48	1,25	112,70
0,08	1,06	0,97	0,89	91,79
0,01	0,94	0,82	0,81	87,26
0,05	2,14	1,78	1,73	83,18
0,05	1,20	1,16	1,11	96,72
0,10	1,08	0,85	0,74	78,05

Online resource 1: Detection accuracy of standard sample with defined percentages of transgenic TCR expressing CD8 T cells

Six batches of standard samples were thoroughly analysed with respect to expected versus detected percentages of TM+ cells in CD45 and CD3+CD8+ gates. Medium background was subtracted from detected percentages and detection accuracy was determined.

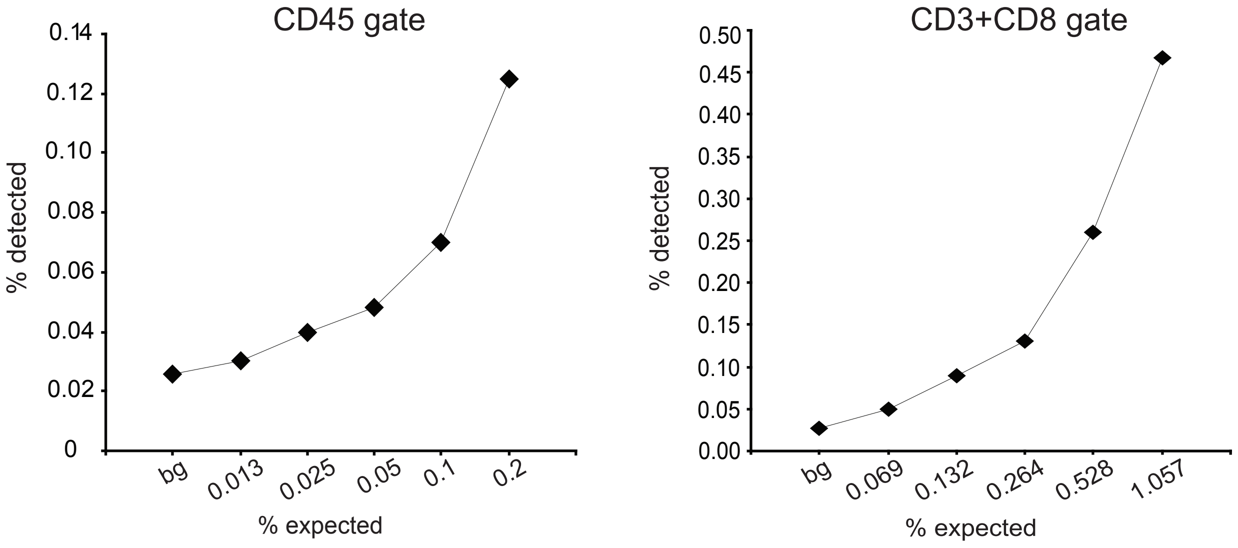

Online resource 2:

The standard sample validates the 2-fold increase in immune response as a solid thumb-rule to define positive responses.

Standard samples were analysed and the detected percentages were plotted versus expected values without subtracting background for CD45 and CD3+CD8 gates.

Data becomes linear at twice the background value.
